# Hub genes in a pan-cancer co-expression network show potential for predicting drug responses

**DOI:** 10.12688/f1000research.17149.2

**Published:** 2019-03-05

**Authors:** Francisco Azuaje, Tony Kaoma, Céline Jeanty, Petr V. Nazarov, Arnaud Muller, Sang-Yoon Kim, Gunnar Dittmar, Anna Golebiewska, Simone P. Niclou

**Affiliations:** 1 Luxembourg Institute of Health (LIH), Strassen, Luxembourg

**Keywords:** co-expression networks, network hubs, drug sensitivity prediction, anticancer drugs, transational bioinformatics, systems biomedicine, biological networks

## Abstract

**Background**: The topological analysis of networks extracted from different types of “omics” data is a useful strategy for characterizing biologically meaningful properties of the complex systems underlying these networks. In particular, the biological significance of highly connected genes in diverse molecular networks has been previously determined using data from several model organisms and phenotypes. Despite such insights, the predictive potential of candidate hubs in gene co-expression networks in the specific context of cancer-related drug experiments remains to be deeply investigated. The examination of such associations may offer opportunities for the accurate prediction of anticancer drug responses.

**Methods:** Here, we address this problem by: a) analyzing a co-expression network obtained from thousands of cancer cell lines, b) detecting significant network hubs, and c) assessing their capacity to predict drug sensitivity using data from thousands of drug experiments. We investigated the prediction capability of those genes using a multiple linear regression model, independent datasets, comparisons with other models and our own *in vitro* experiments.

**Results:** These analyses led to the identification of 47 hub genes, which are implicated in a diverse range of cancer-relevant processes and pathways. Overall, encouraging agreements between predicted and observed drug sensitivities were observed in public datasets, as well as in our *in vitro* validations for four glioblastoma cell lines and four drugs. To facilitate further research, we share our hub-based drug sensitivity prediction model as an online tool.

**Conclusions**: Our research shows that co-expression network hubs are biologically interesting and exhibit potential for predicting drug responses *in vitro*. These findings motivate further investigations about the relevance and application of our unbiased discovery approach in pre-clinical, translationally-oriented research.

## Introduction

The analysis of networks extracted from different types of “omics” data is a useful strategy to enable the characterization and prediction of meaningful properties of the underlying complex biological systems ^1– 3^. Measures of the centrality of genes or proteins in such networks have been shown to be indicators of biological function ^4– 7^. Specifically, the biological significance of highly connected genes, i.e., hubs, in different molecular association networks has been determined using data from several model organisms, molecular interaction types, phenotypes and pre-clinical research applications ^5, 8– 10^. Other research, however, has shown that hub genes in (patient-derived) gene co-expression networks may not have sufficient prognostic value in a few selected classes of cancer ^11^. Despite such insights, the predictive potential of candidate hubs in gene co-expression networks in the specific context of cancer-related drug experiments remains to be thoroughly investigated. An examination of such associations may offer novel opportunities for the accurate prediction and understanding of anticancer drug responses.

Addressing the above-mentioned challenge is now possible thanks to the availability of large collections of data originating from thousands of drug experiments in cancer cell lines. Over the past few years, the investigation of cell line-based computational models for anti-cancer drug sensitivity prediction has been accelerated by publicly-funded efforts of large research consortia. In particular, the Cancer Cell Line Encyclopedia (CCLE) ^12^ and the Genomics of Drug Sensitivity in Cancer (GDSC) ^13, 14^ projects represented significant steps forward for the oncology and pharmacogenomics research communities. These projects have generated genomic and transcriptomic data from thousands of (untreated) cancer cell lines and their accompanying treatment sensitivity measurements for hundreds of experimental and clinically-approved drugs. Using these datasets, computational models for predicting anticancer drug sensitivity based on the analysis of transcriptomic and other types of “omics” data have shown to be useful in the selection and prioritization of candidate compounds for pre-clinical research ^15– 18^.

**Figure 1.**
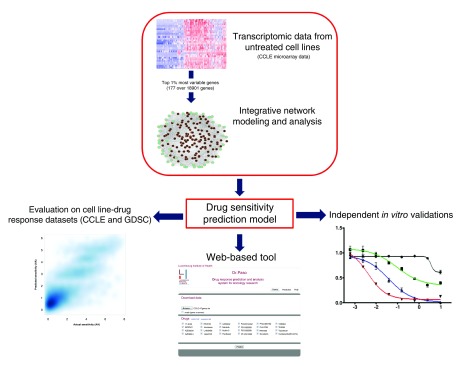
Outline of the research steps, approaches and outcomes of our research.

Here, we investigate the relationship between significant co-expression network hubs and drug responses ( Figure 1). We identified 47 genes representing “hubs” in a pan-cancer transcriptomic network extracted from more than 1000 (untreated) cell lines. These hubs are substantially implicated in a diversity of cancer-related biological processes, and their individual expressions (in the untreated cell lines) are correlated with drug sensitivity. Next, we validated such findings using an independent dataset that also comprises thousands of cell line-drug experiments. We observed that a relatively simple model, based on multiple linear regression, can make predictions that are concordant with the actual drug sensitivity values observed *in vitro*. Moreover, although we do not claim that our model clearly outperforms more complex techniques, its prediction performance is comparable to, and in some cases improves on, previously published models. This is particularly interesting because, unlike prior work, we followed an unbiased discovery approach, i.e.: we did not seek, up-front, a specific set of genes to optimize such a prediction task. 

Motivated by these findings, we used our 47 hub-based model to predict sensitivity scores for four glioblastoma (GBM) cell lines, including three (stem-like) cell lines that were not included in the discovery and validation datasets, against 24 drugs. We selected the top three drugs predicted as highly effective together with a drug predicted as lowly effective (negative control), and performed *in vitro* tests on the 4 cell lines. The sensitivity scores predicted by the hub genes tend to be concordant with the observed *in vitro* responses. Lastly, to facilitate future research, we offer a Web-based interface that allows users to predict drug sensitivity scores for their own samples and expression data with our 47-hubs-based model.

## Methods

### Identification of co-expression network hubs

The published pre-processed CCLE (microarray) gene expression and drug sensitivity datasets were obtained from the CCLE website. In the gene expression dataset, we focused on genes with symbols, calculated their standard deviation (SD) across all samples (1037 untreated cell lines) and ranked them based on their SD. For further analyses, we selected the most variable genes: 177 genes with SD values above the 99 ^th^ percentile of the SD value distribution. The 99 ^th^ percentile was chosen as a stringent data filtering threshold that allowed us to focus on the most highly variable genes in the dataset. This threshold also resulted in a number of genes that was suitable for both computational analysis and post-processing expert interpretations. We computed the gene-gene (Pearson) correlation coefficients between all the 177 genes and merged them into a single gene expression correlation network. We applied WiPer ^19^ to this fully-connected weighted network to detect highly connected nodes (hub genes). This method was selected because: a) it was developed in our team; b) unlike other methods, it offers strict statistical support, i.e., corrected P-values, for each weighted degree value estimated in the network; c) we, and others elsewhere, have previously shown its usefulness for making biologically-relevant predictions ^20– 22^. For each network node, WiPer computes the weighted degree and a corresponding P-value to assess the significance of the observed values, and adjusts it for multiple testing. Genes exhibiting (Bonferroni-adjusted) P<0.05 (100K random network samples for WiPer permutation test) were considered hubs (47 genes) (Dataset 1) ^23^. Drug sensitivity information was not used to select hubs. The resulting 47 genes were examined with different Gene Ontology (GO) and biological pathway analysis tools (below). For each hub gene, we estimated the correlation of its expression profile (across all samples) with the activity area (AA) values available from all sample-drug combinations. The CCLE used the AA as indicator of drug sensitivity. It has been shown that the AA is: a) an accurate estimator of drug efficacy and potency, and b) negatively correlated with the half-maximal inhibitory concentration (IC50), which is an alternative measure of drug sensitivity ^12^. We compared hubs and non-hubs on the basis of such individual expression-sensitivity correlations.

### A drug sensitivity prediction model based on network hubs

We represented each CCLE sample (cell line-drug combination) with the expression values of the 47 hub genes and their corresponding AA values. The full list of CCLE drugs and their annotations are available in the Supplementary Information of 12. We focused on samples with complete expression and AA data. The resulting set of 10,981 (cell line-treatment) samples was used for training and testing regression models. The dataset was standardized by re-scaling each gene so that each gene has mean and standard deviation of 0 and 1 respectively. For each model, we implemented 10-fold cross-validation (CV) for separating training from testing and for assessing prediction performance. We also used leave-one-out CV (LOOCV) and similar prediction performance results were obtained. Diverse regression techniques with different levels of complexity were investigated. We focused on a multiple linear regression model with Ridge regularization (Ridge parameter = 1E-08) because its performance (regression errors) was better than or comparable to those obtained with other techniques, such as support vector machines and k-nearest neighbors, and because of its interpretability in comparison to relatively more complex models. Moreover, we applied ridge regression to achieve a balance between model simplicity, interpretability and prediction power. As in the case of other regularization techniques, by introducing such a ridge penalty, we aimed to reduce the risk of overfitting. Although lasso or elastic net regularizations are also suitable approaches, they would have required the estimation of additional learning parameters and the removal of genes, which were deemed biologically interesting before model training. Moreover, ridge regression allows us to address the problem of multiple collinearity. This is particularly relevant to our research problem as our genes converge to different cancer-related pathways and their expression correlations offer complementary predictive information.

The accuracy of model predictions was assessed by measuring their (Pearson, Spearman and Kendall) correlations with the observed values in the CCLE and the concordance index (CI). The CI approximates, for a random pair of samples, the probability of correctly predicting which sample is more (or less) sensitivity than the other ^24^. A CI equal to 0.5 indicates that the model’s performance is comparable to that from a random predictor, while an index equal to 1 represents the perfect predictor.

### Model evaluations with independent data

Raw expression data were obtained from the ArrayExpress database (accession number E-MTAB-3610) and drug sensitivity (natural logarithm of the IC50 in μM, LNIC50) were downloaded from GDSC database (release 5.0). We normalized raw expression data with the RMA function of the R oligo v.1.42.0 package ^25^. Then we averaged the resulting log2 probe-set intensities to estimate the expression of each gene. Associations between probe-sets and gene symbols were obtained through the hgu219.db v. 3.2.3 annotation package ^26^. For each cell line-drug experiment available (sample), we retrieved the expression data for the 47 genes used as inputs to our prediction model and retrieved the corresponding drug sensitivity values. We focused on the 16 drugs found in both this and the CCLE dataset. This resulted in a dataset consisting of 9,984 samples, each one represented by 47 gene expression values and one LNIC50 value. We standardized expression data as in the case of the CCLE dataset, reformatted the file and input it to the CCLE-derived prediction model (further information below). For each sample in the dataset, the model predicted a drug sensitivity score (approximation of AA). We compared predicted vs. observed values using the indicators applied to the CCLE dataset analysis. We adapted the CI to account for the fact that AA and LNIC50 are expected to be inversely correlated, i.e., for a given sample, concordance is achieved when a high (predicted) AA value corresponds to a low (observed) LNIC50 value, and vice versa.

Access to the CCLE and GDSC datasets, including extensive documentation, are provided in their respective original publications and data websites.

For CCLE RNA-Seq analyses, the RPKM data were downloaded from the CCLE website. Ensembl gene IDs were annotated by gene symbols (GRCh37.69), which were used as unique identifiers. We intersected features (rows) and experiments (columns) of microarray and RNA-Seq datasets and thus obtained two expression matrices of the same size with 16,744 rows and 970 columns. RPKM values of RNA-Seq dataset were additionally log2-transformed: expression = log2(1+RPKM). Next, Spearman correlation was calculated between gene expression profiles corresponding to the same samples. Drug sensitivity prediction model was trained and tested as done with the microarray data. We investigated gene length as a potential source of bias in our analysis as done in 27. As such, we used the maximal transcript length of a gene based on the GRCh37.69 annotation.

### GBM cell lines and expression data for *in vitro* validations

U87 cells, initially obtained from the ATCC (Rockville, USA), were kindly provided by Prof. Rolf Bjerkvig (Department of Biomedicine, University of Bergen, Norway), and were cultured as monolayers in DMEM containing 10% FBS, 2 mM L-Glutamine and 100 U/ml Pen-Strep (Lonza). GBM stem-like cultures (NCH421k, NCH601 and NCH644) were kindly provided by Christel Herold-Mende (University of Heidelberg, Germany) and were cultured as 3D non-adherent spheres as previously described ^28, 29^.

We measured the (baseline) gene expression of four GBM cell lines using GeneChip Human Gene 1.0 ST Arrays (6 U87, 6 NCH421k, 3 NCH644 and 3 NCH601 biological replicates), as reported ^29^. For our model’s 47 genes, we also validated gene expression measurements using quantitative PCR (qPCR) for U87, NCH421k and NCH644 cell lines (each one in triplicate). To this aim, RNA was extracted from 1x10 ^6^ cells using TRI Reagent® (Sigma-Aldrich). RNA isolated in the aqueous phase with a Phase lock gel-Heavy (5 Prime) was precipitated with 100% isopropanol and purified using RNeasy® Mini kit combined with an on-column DNase treatment (Qiagen). For the qPCR, RNA was reverse-transcribed into cDNA using Superscript III™ (Invitrogen) following manufacturer’s instructions. qPCR was performed in 96-well plates using SYBR® Green Master Mix (Bio-Rad) and CFX-96 thermal cycler (Bio-Rad). Normalized gene expression levels were calculated using the CFX manager 3.1 software (Bio-Rad) via the delta-delta Cq method with “Hspcb, Rps13, 18sRNA” as reference genes and taking into account the calculated amplification efficiency for each primers pair. We provide a MIQE-compliance checklist table and details of procedures in Dataset 2 ^23^.

### Drug sensitivity predictions and *in vitro* validation on GBM cell lines

The gene expression dataset was standardized as above. Each sample, represented by a 47-gene expression profile, was input to the prediction model and a drug sensitivity value was predicted for each one of them (18 samples in total), for each of the 24 drugs included in the model. Predicted values were compared between them to determine their relative differences in terms of cell lines and drugs. Next, these predictions were compared to the *in vitro* sensitivity values that were obtained as follows. We tested four drugs: paclitaxel (Sigma-Aldrich), panobinostat, 17-AAG and erlotinib (all Selleck Chemicals) independently on the selected four GBM cell lines with eight drug concentrations (details below and in Dataset 3) ^23^. For each cell line and dose, we performed treatment experiments in triplicate (i.e., 3 treated biological replicates / dose). As a measurement of drug sensitivity, WST-1 (Sigma-Aldrich) cell viability assays were implemented. U87, NCH421k, NCH644 and NCH601 cell lines were seeded into 96-well plates at densities of 1,500, 5000, 4000 and 6000 cells per well, in appropriate culture medium ^29^. Cells were incubated, 24h hours after seeding, with the 8 different drug concentrations ranging from 10 µM to 6.1×10 ^-4^ µM, with a final volume of DMSO not exceeding 0.1% and each condition was tested with six technical replicates. After a 72-h incubation, WST-1 reagent was added in medium to a final concentration of 10%. The adherent cell line (U87) was incubated at 37°C for 2 hours and 3D sphere stem-like cell lines (NCH421k, NCH644 and NCH601) were incubated at 37°C for 6–8 h. Absorbance was measured against a background control at 450 nm on a FLUOstar OPTIMA Microplate Reader (BMG LABTECH). Using the normalized viability measurements, we generated drug dose-response curves and estimated IC50 values (μM) for each sample-drug combination. The dose-response curves were fitted with a four-parameter logistic regression model, whose parameters were calculated using GraphPad Prism 7 (GraphPad).

### Comparisons with other prediction models

We performed multiple comparisons of our hub-based prediction model versus other approaches, including published research. To compare our results with those reported previously ^30^, we implemented an elastic net model. The elastic net model selected has λ and α parameters equal to 0.00105 and 0.95, respectively. The λ value was estimated using the cv.glmnet function (λ value reporting the lowest MSE in a 10-fold cross-validation) in R. The models were trained and tested using 5-fold cross-validation, and were compared on the basis of the CI between the predicted and observed activity areas. To compare our results with those reported previously ^31^, we implemented a SVM using the R package e1071 v. 1.6.8 with default settings excepted for gamma. For this parameter, we used the optimal values determined by Dong *et al*. for each drug ^31^.

LASSO models that optimize drug sensitivity estimation were also investigated. Such models were generated in R using the glmnet v. 2.0.16 package (α = 1). We built models and evaluated prediction performance using a nested CV procedure, and CIs between predicted and observed sensitivity values were reported. We ensured that each of the 10-folds had the same proportion and distribution of sensitivity values for each drug. Within each CV iteration, the cv.glmnet function was used to determine the optimal lambda (using 10-fold CV and based on the minimum RMSE). For model applied to our 47 genes: Optimal λ mean value = 0.0004 (range: [0.00037, 0.0007]).

### Software and web-based tool

We used the R statistical environment for data analysis and visualization ( www.r-project.org), packages: ggplot2 v.2.2.1, pheatmap v. 1.0.10, ComplexHeatmap v.1.17.1 and SNFtool v.2.3.0 ^32^. Concordance indexes ^24^ were calculated based on rescaled Kendall rank correlation coefficients, which were also used to estimate confidence intervals (by Fisher’s transformation). For network analyses, we applied Cytoscape for visualization ^33^, MINE for similarity exploration ^34^ and WiPer for network hub identification ^19^. REViGO ^35^ and g:Profiler ^36^ were applied for biological process and pathway enrichment analyses. The Weka workbench was used for building and testing regression models ^37, 38^, and GraphPad Prism 7 for analyzing drug response curves. A two-tailed, Student’s t-Test was used to estimate statistical differences between correlation values from hubs and non-hub genes. We provide researchers with a Web-based application to enable them to predict anticancer drug sensitivity using their own (47-gene) transcriptomic data (Results). The tool is based on the R Shiny package. Although this package offers useful functionality for generating an interactive user interface, we customized available code using the R Shinyjs package. Users can input pre-processed expression datasets. Alternatively, our application can also implement z-score rescaling of the input data. Figures containing the prediction results can be downloaded and stored as either .png or .jpeg files. Results are also shown as tables with sample-specific predictions (in rows) with their corresponding drugs (in columns), and may be stored as either .csv or .tsv files.

## Results

### Hubs in a pan-cancer transcriptomic network display drug sensitivity predictive potential

**Figure 2.**
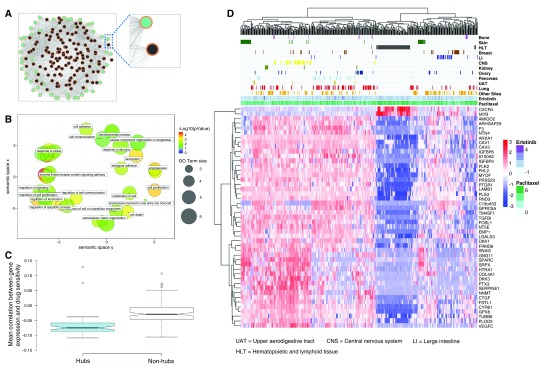
Hubs in a pan-cancer transcriptomic network display drug sensitivity predictive potential. ( **A**) Snapshot of a (fully connected) weighted gene correlation network from untreated cell lines. Nodes and edges representing genes and their correlations respectively. Network hubs and non-hubs are colored in green and black respectively. Nodes are connected by edges, which are depicted in a white-to-grey gradient (the darker the edge, the higher the correlation). A zoom-in view of examples of hub and non-hub nodes reveals that the hub node has more edges with higher weights compared to the non-hub node. ( **B**) Graphical summary of (non-redundant) Gene Ontology terms statistically over-represented in the list of 47 hub genes. Significant Biological Processes terms, represented as bubbles, are projected onto a scatterplot using REVIGO ^33^. Terms sharing common ancestors in the Gene Ontology database are close together; leading to a cluster of GO terms characterizing highly related biological annotations. To facilitate visualization, only a small selection of terms are labeled on the figure. Color and size indicates the term’s level of statistical enrichment in our list of hubs and frequency in the GO database respectively. ( **C**) Comparison of hubs vs. non-hubs on the basis of their individual associations with drug sensitivity (P < 0.0001, two-tailed, Student’s t-test). The boxplot depicts the mean correlation between the gene expression and the Activity Area (AA) values across CCLE cell lines. Box notches indicate 95% confidence interval for each median value. Non-overlapping notches indicates a significant difference at the 95% level. ( **D**) Cell line-drug experiments are visualized in terms of the 47-gene expression data. The panel above the gene expression heat map illustrates the AA values observed for selected sets of cancer cell lines (grouped by tissue site) and two compound examples (erlotinib and paclitaxel) for illustration purposes.

Our hypothesis was that genes highly connected within co-expression networks, i.e., hubs, may be reflective of molecular activity relevant to drug response, across biological processes and tissue sites. To test this hypothesis, we analyzed the CCLE gene expression dataset, which was derived from 1037 (untreated) cell lines representing different cancer types from 18 tissue sites. To reduce network complexity while aiming at preserving potentially relevant information across all samples, we selected genes with highly variable expression pattern across cell lines (i.e., 177 genes with standard deviation of expression values across cell lines located above the 99 ^th^ percentile). Using the pan-cancer expression profiles from these genes, we calculated all the between-gene (Pearson) correlation values and merged them into a fully-connected weighted network ( Figure 2A), which included 177 nodes and more than 15K edges, i.e., correlations (Dataset 1) ^23^.

We identified network hubs by extracting those genes with statistically detectable connectivity scores (i.e., weighted degree values) using WiPer ^19^. This resulted in 47 hubs (WiPer-adjusted P < 0.05, Supplementary Data S1), one of which ( *ANAX1*) is illustrated in Figure 2A together with an example of a non-hub node ( *HCLS1*). A hub is distinguished by the weighted degree, i.e., sum of the edge weights linked to the gene, together with its associated statistical significance (Methods). In Figure 2A, this is in part illustrated by the intensity of the edges (i.e., *HCLS1*’s edges are lighter than *ANAX1*’s edges). The 47 hub genes are significantly implicated in a wide diversity of biological processes and pathways of relevance to cancer progression and therapeutic response. They include cell proliferation, death, migration, adhesion, angiogenesis, kinase signaling and the extracellular matrix ( Figure 2B and Supplementary Figure S1 in Dataset 3) ^23^.

We also investigated the connections between the enriched biological processes ( Figure 2B; GO terms) and known drug targets. Genes associated with a particular GO term were matched to known drug targets annotated in the DGIdb database ^39^. We found that within each biological process term, different genes are known targets of different drugs, though the majority of them are not known to be targets for the drugs investigated here (Supplementary Figure S2 in in Dataset 3) ^23^. We did not find validated evidence that our hub list contains known drug targets. Using DGIdb, we found potential associations between 4 hubs and 2 drugs: DKK1 (with Irinotecan), MYB, SPARC and TUBB6 (the latter three with paclitaxel). However, these associations cannot be interpreted as drug-target interactions and require further investigation.

A GO enrichment analysis of all the genes in the network reported a larger number of statistically enriched GO terms in comparison to the analysis focused on the 47 hubs (biological processes: 196 vs. 74 terms). This may be explained by the increase in the number of genes analyzed. Both gene sets shared in common several significantly enriched processes, including: cell adhesion, proliferation and death. However, there are biological processes that were statistically overrepresented in the 47 hubs exclusively, including endocytosis and several processes specialized in responses to different biological stimuli. These results underscore the significant implication of the 47 hubs in a wide range of cancer-relevant biological processes.

Next, we analyzed the drug sensitivity data (activity areas (AA)) available for these cell lines (11670 cell line-drug experiments) in the CCLE. The AA, which is inversely correlated with the IC50, was defined by the CCLE to approximate the efficacy and potency of a drug simultaneously ^12^. We stress that such data were not considered during the network generation and analysis steps outlined above. For each gene in the network, we calculated the correlation between gene expression and AA across all available (cell line-drug) data, and observed that: a) the expression of hub genes tend to be anti-correlated with drug sensitivity, and b) although such correlations are weak, they are stronger than in the case of non-hub genes ( Figure 2C, P < 0.0001, two-tailed, Student’s t-Test). The 47 hub genes did not include previously reported markers of drug sensitivity, e.g., *ALK*, *BRAF*, *ERBB2*, *EGFR*, *HGF*, *NQO1*, *MDM2*, *MET* and *VEGFRs* ^12, 40^. A possible explanation is that our discovery strategy was not oriented or biased to specific drugs or target families. Moreover, different genes may be associated with a specific drug response without actually representing known targets for the drug.

To further illuminate the information encoded by the 47 hubs, we clustered the samples (available cell line-drug experiment data) based on their (baseline) expression profiles ( Figure 2D). Although, this analysis is based on a simple hierarchical clustering technique and the genes do not clearly segregate all samples in terms of drug responses, these results illustrate the heterogeneity of gene expression profiles and motivated us to further investigate their predictive potential. Using an alternative visualization and (unsupervised) clustering technique, a similar observation could be made (Supplementary Figure S3 in Dataset 3) ^23^. Overall, these results suggest that our 47 hubs represent a novel, biologically meaningful gene set with drug sensitivity prediction potential.

### Predicting drug sensitivity based on network hubs

**Figure 3.**
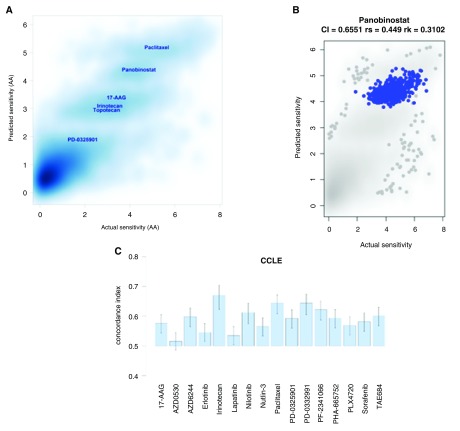
Different views of our model’s predictive capacity on the CCLE dataset using alternative performance indicators. ( **A**) Density plot of predicted vs. actual sensitivity values (n=10981). Pearson, Spearman and Kendall correlation coefficients: 0.86, 0.73 and 0.54, respectively. ( **B**) Focused view of the predicted vs. actual sensitivity for panobinostat, one of the drugs displaying the highest (actual and predicted) activity area (AA) values. Additional examples in Supplementary Figure S5. ( **C**) Concordance indices between the predicted and the observed AA values for a selected set of drugs. An index value = 0.5 is the expected value from random prediction. Error bars: 95% confidence interval of the estimated concordance index.

We used the expression values from the 47 network hubs and drug sensitivity data (n = 10,981, untreated cell line-drug experiments, i.e., samples, with full expression and AA data available in the CCLE) to generate a drug sensitivity prediction model based on multiple linear regression. For a given sample (47-gene expression profile) and drug (identity of one of the 24 CCLE drugs), the model estimates a sensitivity score that approximates the AA values observed in the CCLE. For model training and testing, we used separate datasets respectively through a 10-fold cross-validation sampling procedure. Prediction capability was evaluated with multiple performance indicators that compare the predicted and observed sensitivity values: Pearson, Spearman and Kendall correlations, and a concordance index (CI) ( Figure 3). The R code specifying our prediction model is available on Zenodo ^41^.

Figure 3A and Supplementary Figure S4 (Dataset 3) ^23^ show that the predicted and actual AA values are positively correlated (Pearson, Spearman and Kendall, correlations coefficients: 0.86, 0.73 and 0.54 respectively). In Figure 3A, it is also possible to distinguish a number of clusters that are linked to several drugs with different observed (and predicted) drug sensitivities (Supplementary Figure S5 in Dataset 3) ^23^. For example, the cluster located on the top-right of the plot corresponds to Paclitaxel, followed by a cluster associated with panobinostat, and a third cluster consisting of a mixture of samples tested with 17−AAG, Irinotecan and topotecan. Interestingly, we observe that drugs belonging to the same drug class tend to cluster together according to their predicted (and observed) drug response values. For example, samples treated with cytotoxic drugs (e.g., Irinotecan and Topotecan) and kinase inhibitors (e.g., AZD6244 and RAF265) are closely located on the observed vs. predicted sensitivity plot (Supplementary Figure 5). Figure 3B includes a focused view of the predicted vs. actual sensitivity for panobinostat, one of the drugs displaying the highest (observed and predicted) AA values. This plot and others in Supplementary Figure S5 (in Dataset 3) ^23^ indicate that there are drugs for which our model can make relatively accurate sensitivity predictions in comparison to other drugs in this dataset.

To provide further insights into our model’s prediction capacity, Figure 3C displays the CI for a selected set of drugs. For a random pair of samples, the CI estimates the probability of correctly predicting the relative sensitivities of such samples (e.g., sample X is more sensitive than sample Y) in relation to the observed relative sensitivities. Perfect and random prediction performances are indicated by concordance indices equal to 1 and 0.5 respectively. Our model reported concordance indices with median values above 0.5. Altogether, these results suggest that our 47 hubs are linked to drug responses *in vitro*, and that their predictive potential deserves further investigation.

### Hubs and their drug sensitivity associations are measurement-platform independent

We compared our results to those obtained from the CCLE’s RNA-Seq dataset, which was made publicly available last year. First, we investigated the similarity of the (original) microarray and RNA-Seq datasets and observed a high level of concordance between these datasets, with mean Spearman correlation between gene expressions profiles of 0.87 (confidence interval at 95%: 0.870–0.871). The correlations for our network hubs was even higher: 0.94 (global expression of 47 genes among all cell lines) (Supplementary Figure S6 in Dataset 3) ^23^. Also we generated a mean-standard deviation representation of the genes characterized by both techniques (Supplementary Figure S7 in Dataset 3) ^23^. In both platforms, the 47 genes show high variability and moderate average expression, and none of them was lowly expressed. These observations indicate the inter-platform robustness of the network hubs in terms of their gene expression.

We also investigated the predictive performance of our model when RNA-Seq data were used instead of microarrays. The overall prediction performance obtained in both application scenarios was almost identical. CI: 0.772 vs. 0.770, and Spearman correlation: 0.728 vs.0.725 (microarray and sequencing data respectively). Lastly, we further compared the connectivity of our 47 genes in networks generated with data from the two platforms independently. We regenerated gene networks based on microarray and sequencing data, and this time considered the sum of R ^2^ as a measure of degree of each gene (node) and visualized the distribution for all genes and the 47 hubs. We observed that, in both platforms, our 47 genes are shown as top hub genes (Supplementary Figure S8 in Dataset 3) ^23^. These analyses corroborate the robustness of the gene expression profiles and predictive properties of our hub-based signature in microarray and RNA-Seq platforms. Also we assessed the length of the genes in our signature and found that 42 of 47 genes were longer than 2000 nt. Based on our previous experience ^27^, we should not expect negative effects switching from arrays to sequencing for the vast majority of the genes.

### Assessment of drug sensitivity prediction potential on an independent dataset 

**Figure 4.**
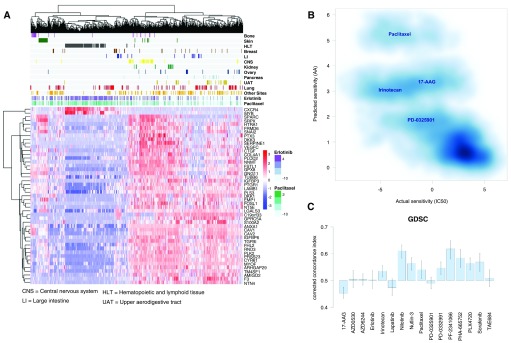
Different views of our model’s prediction capacity on the GDSC dataset. ( **A**) Cell line-drug experiments are visualized in terms of the 47-gene expression data. The panel above the gene expression heat map illustrates the natural logarithm of half-maximal inhibitory concentration LNIC50 (μM) values observed for selected sets of cancer cell lines (grouped by tissue site) and two compounds (erlotinib and paclitaxel). ( **B**) Application of CCLE-derived model to the GDSC data. Density plot of predicted (activity area (AA)) vs. actual sensitivity (LNIC50) values for drugs that are common between the CCLE and GDSC (n = 9,984). Pearson, Spearman and Kendall, correlations coefficients: -0.72, -0.71 and -0.50 respectively. ( **C**) Concordance indices between the predicted and the observed sensitivity values. An index value = 0.5 is the expected value from random prediction. Indices are corrected to account for the notion that higher concordance is reached when high AA (prediction) corresponds to a low LNIC50 (observed) values, and vice versa. Error bars: 95% confidence interval of the estimated concordance index.

We tested the drug sensitivity prediction capacity of our 47 hubs on the 2016 release of the GDSC dataset, which partially overlaps with the CCLE in terms of cell lines and drugs ^42^. To allow our CCLE-derived model to make predictions on this dataset, we focused on the 16 drugs that are found in both datasets. First, as in the case of the CCLE data, we show that the (baseline) expression profiles of these 47 genes are diverse across samples and drugs ( Figure 4A, and Supplementary Figure S3 in Dataset 3) ^23^. Note that in the GDSC dataset drug sensitivity is represented as the logarithm of IC50 (LNIC50) values (AA values were not provided in this dataset).

Next, we applied our (CCLE-derived) prediction model to the GDSC data and made sensitivity predictions (AA values) for all the samples (cell line-drug experiments) available (Methods). The resulting predictions were then compared with the actual sensitivity values in the GDSC dataset ( Figure 4B, and Supplementary Figure S4 in Dataset 3) ^23^. As required, the predicted (AA) and actual sensitivity (LNIC50) values for these samples (n = 9,984) are anti-correlated (Pearson, Spearman and Kendall, correlations coefficients: -0.72, -0.71 and -0.50, respectively). This indicates that our 47-hub-based model is, in general, estimating sensitivity values that are in agreement with those observed in a test dataset, i.e., higher predictive agreement is reached when high AA (prediction) relates to a low LNIC50 (actual) values, and vice versa.

Figure 4C summarizes the assessment of our model’s predictive performance on the GDSC dataset based on (drug-specific) CIs, as done for the CCLE dataset ( Figure 3). Concordance indices > 0.5 were obtained for 12 out of the 16 drugs, and (among those 12 drugs) concordance estimates for 9 drugs can be reliably interpreted as larger than 0.5 (95% confidence intervals of the estimated indices). The predictive performances for several drugs (e.g., Nilotinib, Nutlin-3 and Sorafenib) are very similar to those estimated in the CCLE dataset. As in the CCLE dataset, the sensitivity observed in samples treated with AZD0530 and Lapatinib proves to be more difficult to accurately predict. Although concordance indices > 0.5 were obtained for irinotecan and paclitaxel predictions, this represents a reduction of prediction performance in comparison to the predictions made for CCLE samples. The prediction performance of 17-AAG, PD-0325901 and TAE684 were also diminished. Overall, our findings further suggest that our network hubs are relevant for predicting drug sensitivity, and highlight challenges in a drug-specific context.

### Further evaluations and comparison with alternative modeling approaches

As the GDSC dataset shares cell lines in common with the CCLE, we also assessed the prediction performance of our hub-based prediction model on GDSC cell lines that are not included in the CCLE. To do this, we applied our CCLE-based model on the GDSC dataset and made a distinction between predictions for overlapping and unique cell lines. When focused on experiments with cell lines found in both data sets, we obtained the following correlations between predicted (AA) and observed sensitivity values (IC50): -0.73 (Pearson), - 0.72 (Spearman) and -0.52 (Kendall). For cell lines uniquely represented in the GDSC, we obtained the following correlations: -0.72, -0.68 and -0.48. Although a slight reduction in prediction performance is observed, these results are comparable and stress the robustness of our prediction results for different types of cell lines, including those not included in our hub discovery and model training dataset.

Also we investigated the stability of hubs across the CCLE and GDSC datasets. To do this, we repeated the network generation and hub identification procedures on the GDSC with WiPer (Methods). This analysis resulted in the detection of 69 network hubs (as before, WiPer adjusted P-value < 0.05). Among such genes, 23 genes are also found in our 47-gene signature, such as: *VEGFC*, *CAV2*, *MYOF*, *CAV1* and *TM4SF1*. Although this overlap does not include the full set of hubs obtained in our CCLE analysis, it gives an indication of the robustness of a set of such genes despite the important differences between the datasets in terms of the numbers and types of cell lines.

To further demonstrate the robustness of our predictions, we implemented multiple runs (or iterations) of the 10-fold cross-validation (CCLE data) and assessed their reported performances. For 100 independent (10-fold) cross-validations, the prediction performance is very similar: all iterations reporting CIs between 0.765 and 0.77, and a coefficient of variation = 0.026% (Supplementary Figure S9 in Dataset 3) ^23^. 

Using our 47-hub signature, we also investigated (multiple linear regression) models trained on the GDSC and tested on the CCLE datasets. Although comparable with the cross-validation results obtained with the CCLE dataset, the GDSC-based cross-validation showed an overall improvement in drug sensitivity prediction performance: CI = 0.82, rs = 0.82 and rk = 0.64 (Supplementary Figure S10 in Dataset 3) ^23^. Next, we applied the resulting model to the CCLE dataset. The prediction performance is similar to that obtained with the CCLE-derived model (Supplementary Figure S11 in Dataset 3) ^23^. Moreover, as in the CCLE-derived model, we observed that the predictive quality is relatively higher or deteriorated according to specific drugs.

We also investigated the impact of reducing the 47-gene set on prediction performance. We used our 47 genes as inputs to LASSO modeling, and we observed that is possible to generate models with an average of 44.6 genes (range: 43 to 46 genes). However, LASSO-based models offered very similar prediction performance in comparison with our 47-gene model (CCLE, using a nested 10-fold CV, mean CI: 0.77 ± 0.004).

We also implemented a drug sensitivity prediction model based on LASSO using all gene expression features as inputs to the model. The resulting model consisted of 605 genes, which did not include any of our 47 hubs. When comparing the prediction performance of our 47-gene model vs. the 605-gene LASSO model, we did not observe significant differences, though the latter offered a slightly higher prediction performance (CCLE, nested 5-fold CV, CI: 0.77 vs 0.80). This relative improvement in performance is not surprising as the LASSO model, unlike our hub discovery strategy, explicitly sought to identify the best set of genes for optimizing this specific regression task.

To assess the effect of network size on the identification of hubs, we applied our hub detection analysis to a larger network consisting of 530 genes. These genes were selected with a more flexible filtering criterion (Methods): Genes showing SD of expression above the 97th percentile of the SD value distribution. As expected, a larger number of significant hubs were detected in this network (203 hubs, at corrected P-value < 0.05). Among them, our original set of 47 hubs were included, which reiterates their statistical significance and robustness of our analysis. This was also observed when repeating the analysis using a far less stringent procedure for estimating statistically significant hubs, i.e., P-value estimation. Using only 1000K permutations to estimate P-values, we detected 212 candidate hubs (corrected P-values < 0.05) that also included our original set of 47 hubs.

### Comparisons with published prediction models

We re-implemented models previously reported ^30, 31^, and compared their performance with our model. We chose these works because of their model coverage and analytical depth using different supervised prediction techniques. However, note that unlike our discovery strategy, their models were based on input genes that were explicitly sought to optimize drug sensitivity prediction. Also, unlike our model, Dong *et al*. ^31^ considered prediction of drug sensitivity as a classification problem. Given a gene expression dataset, their approach aimed at assigning each sample to one of two pre-established response classes: resistant and sensitive. They used CCLE data to build their models. For each drug, they started by discretizing a “scaled AA” (sAA) into three categories: resistant if sAA < -0.8 SD (standard deviation which is equal to 1), sensitive if sAA > 0.8 SD and intermediate otherwise. After removing samples with an intermediate response, they focused on the classification of the extreme response classes (resistant vs, sensitive). Their drug-specific models were based on a support vector machine (SVM) and recursive feature selection using gene expression data. They reported an accuracy of 0.81 (on average) when their models were cross-validated on the CCLE. The performance was considerably reduced when tested on GDSC data (only 3 out of 11 drug models reported an average AUC equal to or above 0.69).

Therefore, to directly compare Dong *et al*.’s models with ours, we had to re-specify and re-implement our drug sensitivity prediction approach. This is needed because our approach is defined as a regression problem and is not constrained to predetermined sensitivity classes. Hence, we first labeled the samples as sensitive and resistant as done by Dong *et al*. We then tested whether the predicted sensitivities (predicted AA values from our model) correctly assign each sample to the “right” sensitive and resistant classes. The predictive performances of our and Dong *et al*.’s models are comparable with a small advantage for Dong *et al*.’s models (average AUCs = 0.79 vs. 0.73, Supplementary Figure S12 in Dataset 3) ^23^. However, this advantage is not surprising since Dong *et al*.’s models optimizes the separation of two well-distinguished sensitivity classes. Our predictions are obtained from a regression model trained and tested on all samples with all available sensitivity values. Despite such a caveat, the prediction performance achieved by our 47-hub model was very similar to the performance from Dong’s drug-specific models except for five drugs (AZD0530, erlotinib, lapatinib, LBW242, PD-0325901) out of 21 models (drugs), and our model clearly outperformed their model for one drug (PD-0332991).

In the comprehensive study by Jang *et al*. ^30^, thousands of models were compared and the authors concluded that an elastic net-based model was the best choice. Therefore, we trained and tested an elastic net model, and compared it to our model. The models were trained and tested using 5-fold cross-validation, and were compared on the basis of the concordance between the predicted and observed activity areas. The elastic net model, overall, outperformed our 47-hub model (average CI of 0.81 vs. 0.77). However, the elastic net model required 614 genes as input features to achieve this performance (with no genes in common with the 47 hub genes). As the difference in concordance between these models was only 0.04 on average, we also compared the individual predicted sensitivity values generated by the two models. We found that their predicted sensitivity values are highly correlated (0.99 of correlation and average difference of 0.02). These results, which are graphically illustrated in Supplementary Figure S13 (in Dataset 3) ^23^, indicate that these models’ prediction performances are comparable.

Additionally, we implemented prediction models based on the gene expression of well-known markers for drugs used in clinical practice, and which were also included in our datasets. Here we report results for two such markers: PDGFR (a target of Sorafenib) and EGFR (and target of Erlotinib), which were used as inputs to prediction (linear regression) models. To make an unbiased comparison, we compared prediction performances specific to each drug. For both drugs, we found that models built with our 47 hub genes outperformed models built with the gene expression of these targets. For erlotinib, our model reported a CI = 0.62, while the EGFR-based model showed a CI = 0.57. The difference was more significant for sorafenib: Our model reached a CI = 0.57, whereas models built with either *PDGFRA* or *PDGFRB* reported CIs below 0.5 (0.48 and 0.47 respectively).

### Independent *in vitro* validation

To further validate the prediction potential of our network hubs on independently-generated data, we made predictions and performed *in vitro* tests for several GBM cell lines and compounds in our lab. First, we measured the (baseline) expression profiles of four (untreated) GBM cell lines that have been well-characterized in our lab: U87, NCH644, NCH601 and NCH421k. While the CCLE and GDSC datasets included U87, the latter three are stem-like GBM cell lines that were not included in our previous analyses.

Although genome-wide expression data can appropriately cluster multiple samples (biological replicates) from such cell lines, we found that the expression profile of our 47 genes are sufficient to achieve the same biologically-meaningful segregation while offering a clearer, fine-grained view of their differences (Supplementary Figure S14 in Dataset 3) ^23^. We also verified the platform-independent replicability of these results with another 47-gene expression dataset derived from three of these cell lines measured with qPCR (Supplementary Figure S14 in Dataset 3) ^23^. These results show the biologically-relevant discriminatory capacity and reproducibility of our 47-hub expression profiles in our set of brain cancer cell lines using microarrays and qPCR. Raw qPCR Cq values are available on Zenodo ^23^.

**Figure 5.**
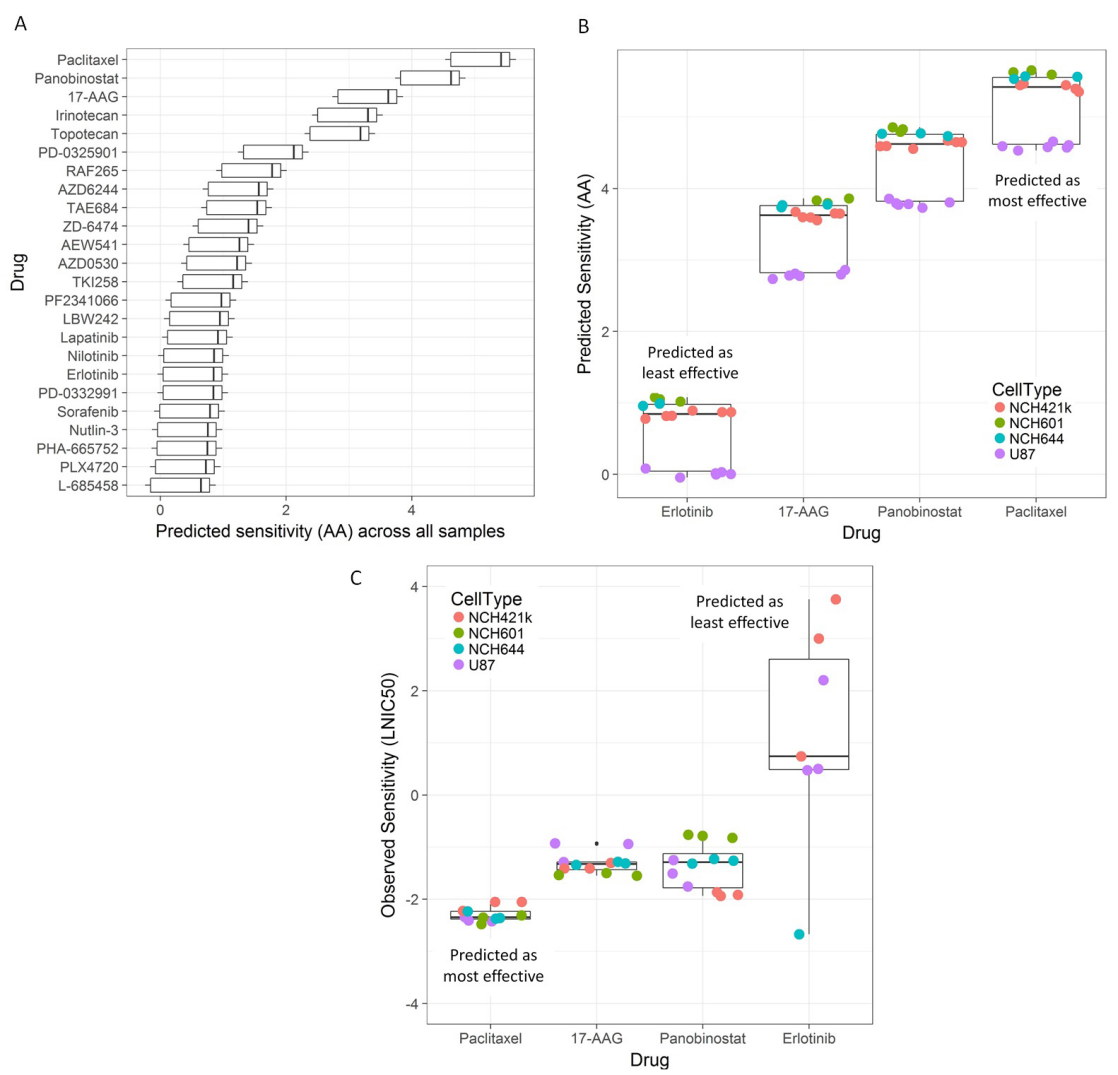
Drug sensitivity predictions and *in vitro* validation for different glioblastoma cell lines and compounds. ( **A**) Sensitivity predictions (horizontal axis) for 24 drugs (vertical axis). Box plot summarizes the (432) predicted sensitivity (activity area (AA), as defined in the prediction model) values for four glioblastoma cell lines: U87, NCH644, NCH601 and NCH421k. Only the U87 cell line was included in the model learning phase. The 47-gene expression profiles of multiple biological replicates (18 samples in total) were input to the prediction model (six U87, three NCH644, three NCH601 and six NCH421k samples). ( **B**) Alternative boxplot summary of the prediction results for four drugs (erlotinib, 17-AAG, panobinostat and paclitaxel) and the different cell lines. These drugs, which were selected for subsequent *in vitro* tests, were predicted to be relatively highly (17-AAG, panobinostat and paclitaxel) and lowly (erlotinib) effective against the four cell lines. ( **C**) Summary of *in vitro* test results. The selected drugs were tested on each cell line in triplicates, relative viability (vs. vehicle-treated samples) was measured for eight drug concentration values (µM) and half-maximal inhibitory concentration (IC50) values were estimated for each drug-sample experiment. The boxplot shows the resulting natural logarithm of IC50 (LNIC50) values obtained. Drug response data for NCH601 samples and erlotinib are not available, and for NCH644 samples and erlotinib not shown because of lack of effect. Boxes show the median, the 25 ^th^ and 75 ^th^ percentiles (lower and upper hinges), and (1.5x) interquartile ranges.

Next, our model predicted the sensitivity of the four GBM cell lines (18 samples in total, Methods) against the 24 drugs included in the model. The baseline 47-hub expression profiles of these cells were input to the prediction model (six U87, three NCH644, three NCH601 and six NCH421k gene expression profiles). Figure 5A summarizes the 432 predicted sensitivity (AA) values according to drug (18 predictions per drug). To investigate such predictions *in vitro*, we focused on the top-3 drugs associated with the highest predicted sensitivities (paclitaxel, panobinostat and 17-AAG), as well as on erlotinib, which was predicted as an ineffective compound. The main reason for the selection of these compounds was our interest in investigating compounds predicted to be highly active (3-top drugs) together with a “negative” control that was predicted, and expected, to have lower activity (Erlotinib). Moreover, these drugs correspond to four different drug classes: cytotoxic, histone deacetylase inhibitor, antibiotic derivative and an EGFR inhibitor respectively. In the case of erlotinib, the predictions are consistent with the fact that the tested cells do not (NCH644, NCH421k) or very lowly (U87, NCH601) express EGFR. Figure 5B and Supplementary Figure S15 (in Dataset 3) ^23^ show a more focused view of the predicted sensitivity values for our samples against these four drugs.

We tested the selected drugs on each cell line, in triplicates, and measured their response based on their relative viability (i.e., normalized to vehicle-treated samples) for eight drug concentration values (µM). For each treated cell line, we estimated the IC50 values and compared them on the basis of cell line and drug groups. Figure 5C summarizes the results with boxplots showing the LNIC50 values. Drug response data for NCH601 samples and erlotinib were not available (not tested), and data for NCH644 samples and erlotinib are not shown due to lack of effect. Supplementary Figure S16 (in Dataset 3) ^23^ includes all the drug response curves and additional details.

As predicted by our model, all our cell lines exhibited the lowest sensitivity, i.e., the highest IC50 values, when treated with erlotinib (median LNIC50 = 0.74 µM). Overall, U87 tended to be the least sensitive cell line in relation to all four drugs (median LNIC50 = -1.27 µM across all sample-drug experiments), though it did not show the lowest sensitivity for every single compound or biological replicate. Our model consistently predicted NCH601 as the most sensitive cell line against all drugs (Supplementary Figure S15 in Dataset 3) ^23^. Our *in vitro* tests showed that NCH421k tends to be more sensitive than NCH601 (median logIC50: -1.64 vs. -1.54 µM). Despite this particular discrepancy, we found a global agreement between predicted and observed sensitivities on the basis of cell type (Spearman correlation between the median sensitivity values, predicted (AA) vs. observed (LNIC50) in the four cell line groups: -0.40).

In accordance with the predictions, Paclitaxel was the most effective drug across all treated samples (median LNIC50 = -2.35 µM). Lesser agreement between predicted and observed sensitivities were obtained in the case of the remaining two drugs. For all samples, our model predicted overall higher sensitivity for panobinostat than for 17-AAG ( Figure 5B). Relatively similar responses were obtained, *in vitro*, for panobinostat (median LNIC50 = -1.29) and 17-AAG (median LNIC50 = -1.33 µM), though a larger variability of sensitivity values was observed in the former case. Nevertheless, predictions and *in vitro* tests concordantly showed that NCH421k and U87 samples treated with panobinostat were consistently more sensitive than all samples treated with 17-AAG ( Figure 5C and Supplementary Figure S16 in Dataset 3) ^23^.

We had a closer look at topotecan, a drug that may be expected to exhibit differential activity for at least one (but not all) of the cell lines investigated. This drug is known to target TOP1 (DNA Topoisomerase I). In our set of GBM cell lines selected for validation, TOP1 is relatively highly expressed in NCH601 and weakly expressed in U87. Moreover, this target is not included in our 47-gene signature. As illustrated in Supplementary Figure S17 (Dataset 3) ^23^, our model predicted relatively higher sensitivity values for NCH601 than for U87. Furthermore, Topotecan is predicted to be more effective than Erlotinib in all 4 cell lines. Taken together, these results provide further evidence of the potential of our 47-hub-based model for predicting drug sensitivity *in vitro*, and will encourage future investigations.

### Dr.Paso online

**Figure 6.**
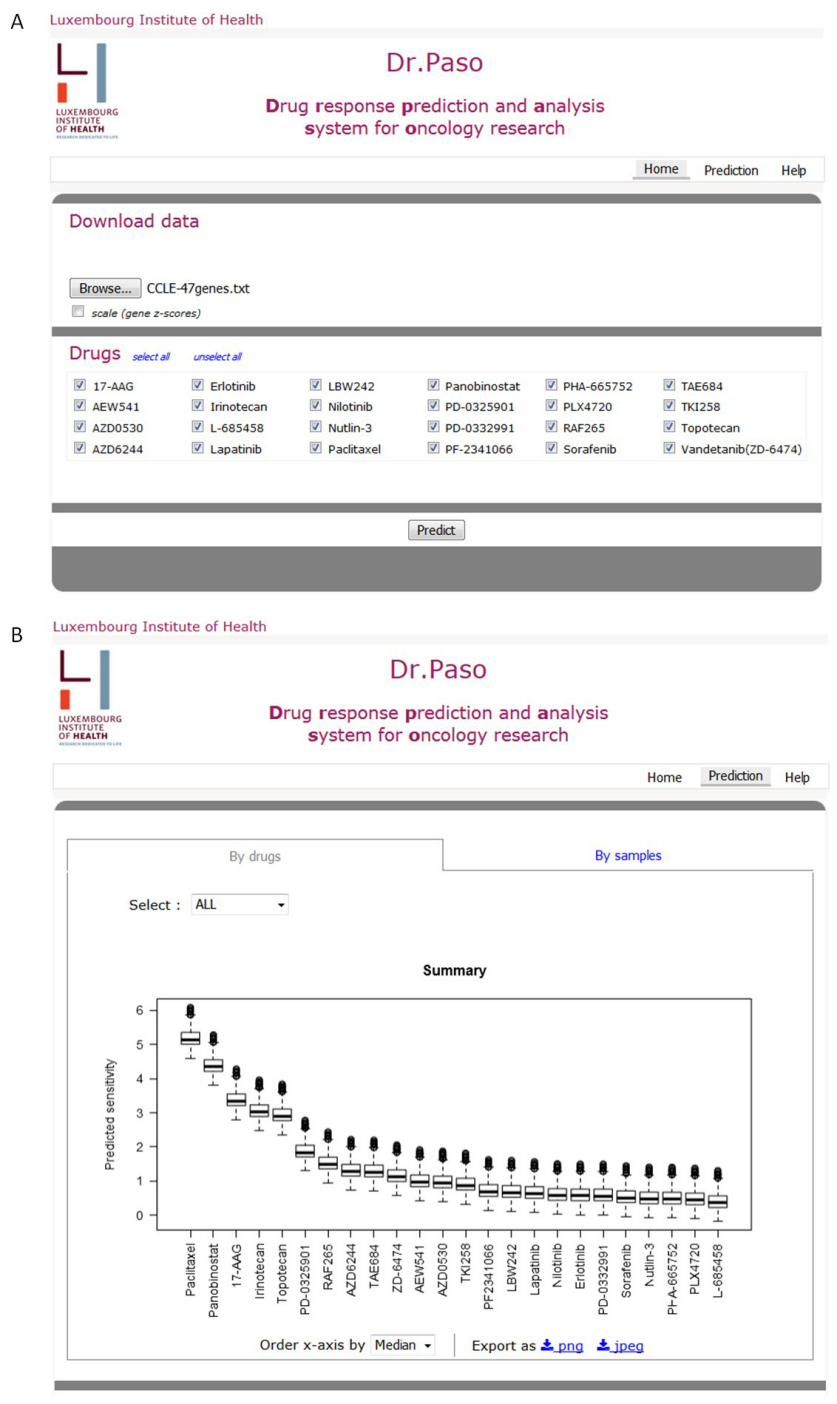
Dr.Paso online: a Web-based tool for predicting drug sensitivity and enabling further research. Screenshots of: ( **A**) Main page with user input and analysis options; ( **B**) Global view of predicted sensitivity values for a given input gene expression dataset and all drugs available in the CCLE; (C) Alternative view of predictions focused on a specific input sample and all drugs; (D) Tabular-based view of results. All views can be selected and downloaded according to user requirements.

To enable further research, we developed a web-accessible tool that allows researchers to upload their own gene expression data, make sensitivity predictions and visualize results in a few steps ( Figure 6). We term this tool: Dr.Paso (Drug Response Prediction and Analysis System for Oncology Research) ^41^. The Help section of the website offers a guided application example using CCLE data. Users provide their input data as a text file containing the (baseline) 47-gene expression for different samples, and then can select all or specific drugs for making predictions ( Figure 6A). Dataset re-scaling (feature standardization with means and standard deviations equal to 0 and 1, respectively) can be applied to harmonize the input dataset with the feature representation used in our model. Prediction results are presented with graphical displays and tables in different panels. Moreover, users can control the amount and focus of information at the drug and sample levels ( Figures 6B–D). Results can be saved in different graphical and tabular file formats. The tool is freely available at www.drpaso.lu.

## Discussion

The biological relevance of hubs in different types of molecular networks has been previously investigated, e.g., in the context of gene lethality. The predictive potential of candidate hubs in gene co-expression networks in the specific context of cancer-related drug experiments deserve deeper investigations. This is important not only for further understanding the biological roles of network hubs, but also because such knowledge may offer new opportunities for the accurate prediction of anticancer drug responses. Here we investigated the relationship between hubs detected in a pan-cancer co-expression network and drug sensitivity *in vitro*.

The development of computational models for estimating drug sensitivity based on gene expression data from large collections of cancer cell lines is important to support pre-clinical research, and provides a basis for future clinically-oriented applications. Our research offers insights into such challenge through the integration of network-based and statistical modeling approaches. For a given drug, we showed that in principle it is possible to predict anti-cancer drug sensitivity based on the gene expression profile of 47 genes, which represent significant hubs in a pan-cancer transcriptomic network and are prominently implicated in a variety of cancer-relevant biological processes. This is particularly appealing because at the start of our investigation we did not aim to select a specific set of genes that could optimize the supervised prediction of drug sensitivity. We implemented an unbiased discovery approach, which was motivated by the hypothesis that co-expression network hubs encode useful information for investigating drug response *in vitro*.

The prediction model resulting from our network hub analysis is not proposed as a competitor for existing approaches for drug sensitivity prediction. Nevertheless, our study and other previous research highlight the challenges and complementary predictive capacity exhibited by different modeling approaches ^15, 43^. No single model can consistently make accurate predictions for all drugs and cell lines available in the CCLE and GDSC datasets, including models that include genomic data and more complex learning parameters ^30, 44, 45^. Different models can offer more, or less, accurate predictions for certain drugs, and there is no conclusive evidence about the dominance of a particular modeling technique ^46^. For example, our model makes good predictions for irinotecan, panobinostat and PF2341066, all of them with AUC > 0.85 and CI > 0.6. Moreover, these examples are highly comparable with the performance obtained by previous work, e.g., in 31. Also in comparison to Dong *et al*. ^31^, our model made more accurate predictions for PD-0332991 (AUC=0.84 vs. 0.75), but weaker predictions for lapatinib (AUC = 0.62 vs. 0.74). Such limitations may be partially explained by a lack of sufficient molecular information to account for the complexity of cell lines and their drug responses, choice of surrogate measures of drug sensitivity and inconsistencies of sensitivity data between the CCLE and GDSC ^40, 47, 48^. The latter may also partly explain the overall degradation of predictive performance when training models on the CCLE and testing them on the GDSC.

The predictive capacity of our 47-hub model is grounded in an unbiased network-guided selection of model inputs prior to the fitting of a regression model. Future investigations, motivated by new datasets and clinically oriented questions, are certainly envisaged and are expected to include new biomarker discovery and prediction modeling strategies. There is a need, for example, for additional research on the connection between network hubs and drug sensitivity with a focus on particular cancer types or drugs. Our analyses indicate that on the basis of tissue sites, the top-3 cancer types for which our model makes relatively highly accurate predictions are: thyroid, pancreas and prostate cancers, with CIs = 0.8, 0.86 and 0.86 respectively (CCLE data and using 10-fold cross-validation). Predictions for breast-derived samples reported lower performance (CI = 0.74). Importantly, although gene expression profile of hematopoietic and lymphoid samples differ from all other samples, our 47-hub model was able to predict their responses with a relatively good accuracy (CI = 0.75). Our investigation was limited to the drugs available in two well-established datasets. As larger collections of data from drug experiments become publicly available, it will be possible to develop more extensive analyses for newly approved or experimental compounds. Although we provided evidence of the robustness of our analyses when using microarray, RNA-Seq and qPCR data, the impact of expression data generation platforms on drug sensitivity prediction deserves further research. Also the analysis of larger networks, including those generated using different data filtering methods, is an interesting topic that deserves future research.

Here, we focused on gene expression data for two reasons: i) Our network-based biomarker discovery strategy is based on the analysis of gene expression data; and ii) previous research (using CCLE and GDSC datasets) has indicated that, although mutation and copy number alterations can be informative, the most powerful prediction models are those based on gene expression data ^12, 13, 17, 42^. Nevertheless, future work could benefit from the incorporation of other “omics” data types to investigate different types of networks and hubs. Although we did not identify major effects when using the latest version of the CCLE gene expression data (RNA-Seq), future work could include additional analyses and models based on such a dataset. In this article, we started using the microarray version because it was the only gene expression dataset available at the beginning of our project. Future work may also be motivated by the fact that the CCLE RNA-Seq dataset could allow the analysis of transcript-level (gene isoform) data for predicting drug response. Such information has been recently shown to be a useful source of features for drug sensitivity prediction ^49^. Moreover, the investigation of the biological role of hubs in gene isoform networks may open new directions for drug sensitivity research and other applications. Furthermore, there are other opportunities to be investigated such as the analysis of genomic alterations, non-coding RNAs and epigenetic markers, which may enhance or complement existing models for predicting drug sensitivity.

Inconsistencies in drug sensitivity as measured for the same cell lines across different studies, i.e., independent datasets, can also limit the application of insights derived from a single dataset. We expect that in the future we can address this point by either: a) weighing sensitivity values according to the available experimental evidence derived from multiple datasets, b) building global models that can generate predictions in an integrated fashion using multiple, independent datasets, or c) investigating models based on harmonized versions of datasets obtained from different studies ^50^. Another limitation of our study is the use of two drug sensitivity measures, AA and IC50, as provided by the CCLE and GDSC datasets, to assess prediction performance. Further investigations will involve prediction performance analysis based on common measures of sensitivity. Such analyses will, nevertheless, be limited by potential inconsistencies in experimental sensitivity measurements across studies, as reported in the case of the CCLE and GDSC data ^40^. Therefore, future work will require the incorporation of harmonized versions of such and other datasets, such as those recently generated by the PharmacoDB project ^50^.

Beyond a connectivity-centric interpretation of hubs, an interpretation of their potential functional roles in co-expression networks is not straightforward. Based on their implication in different cancer-related biological processes and their high expression correlations with many genes involved in different pathways, it is reasonable to postulate that our 47 hubs may have relevant mechanistic roles in the drug response context. Moreover, we found that these genes are related to different known drug targets via multiple biological processes, which may offer clues about the potential signaling controlling role of the hubs. However, these and alternative interpretations will require further investigations.

Overall, while further investigations are needed, our study offers evidence of the relevance of gene co-expression network hubs in the context of drug sensitivity and cancer research. We hope that our findings will enable deeper investigations and pre-clinical research applications.

## Data availability

### Underlying data

Full qPCR data (including raw Cq values) are available on Zenodo ^23^.

Data are available under the terms of the Creative Commons Attribution 4.0 International license (CC-BY 4.0).

### Extended data

Extended data associated with this study are available on Zenodo ^23^.

Dataset 1. Gene co-expression network data. It contains network nodes, weighted network and list of hubs.

Dataset 2. qPCR data from independent validation, including MIQE and additional information.

Dataset 3. Supplementary Figures. Legends are included under each figure.

Data are available under the terms of the Creative Commons Attribution 4.0 International license (CC-BY 4.0).

## Software availability

**Software available from:** www.drpaso.lu.

**Archived source code at time of publication:** https://doi.org/10.5281/zenodo.1689979 ^41^.

**License**: **MIT license.**
