# Supplementary material for: Hub genes in a pan-cancer co-expression network show potential for predicting drug responses
Source: F1000Res. 2019 Mar 5;7:1906. Originally published 2018 Dec 7. [Version 2] doi: 10.12688/f1000research.17149.2 (PMC6406180; doi:10.12688/f1000research.17149.2)
Supplement: Peer review history

# Peer review: Reviewer response for version 2

**DOI:** 10.5256/f1000research.20113.r45288

**Published:** 2019-03-06

**Authors:** Elizabeth A. Coker

**Affiliations:** 1 Translational Cancer Genomics, Wellcome Trust Sanger Institute, Hinxton, UK

I am satisfied with the revisions the authors have made to the manuscript.

I have read this submission. I believe that I have an appropriate level of expertise to confirm that it is of an acceptable scientific standard.

# Peer review: Reviewer response for version 1

**DOI:** 10.5256/f1000research.18749.r42997

**Published:** 2019-02-15

**Authors:** Therese Commes

**Affiliations:** 1 Universite de Montpellier, Montpellier, France

The authors present an interesting work for predicting cancer cells drug responses based on " *the gene expression profile of 47 genes representing significant hubs in pan cancer transcriptomes" *from untreated cells lines. The approach is original, as they did not select a specific set of genes from drug sensitivity experiments, but proposed " *an unbiased discovery approach,* *motivated by the hypothesis that co-expression network* ** *hubs encode useful information for investigating drug response* ** *in vitro* *"* *. *Next, they investigated co-expression network hubs and drug responses and validated their approach using independant data sets including cell line-drug data.

 The study is well conceived and executed. The approaches used are suitable, and the description of work is adequately detailed. Data are clearly presented, and for the most part conclusions are reasonable. The method appears interesting, I think the paper would be a nice contribution that will be well-cited. Despite the difficulty of comparison, prediction performance of the model was compared to existing published methods and shows comparable results. Moreover the manuscript is well written.

 The authors need to clarify on the following points:

 Figure1:

 It will be better to specify the type of data (microarrays?) and source (CCLE?) in input for *"transcriptomic data from untreated cell lines".*

 Methods, page 4 : **

 The reason why removal of genes is needed is not clear: *"Although lasso or elastic net regularizations are also suitable approaches, they would have required the estimation of additional learning parameters and the removal of genes, which were deemed biologically interesting before model training......."*

 Methods, page 5 (line 24) :

 The authors should specify what they mean by " *the original data"* which kind of files they used (raw data, gene counts ...). (line 28: RNS-seq instead of RNA-seq).

 Results, page 9:

 Could the authors specify if drugs with nearest clusters or CI values belong to a same drug "class" or not? Is there a relationship between performance prediction and drug classes?

 Results, page 9:

 This sentence is not clear: " *As the GDSC dataset shares "different" cell lines in common with CCLE*..."

 Results, page 12:

 " *However elastic net model required 613 genes as input features"*: Does this set include the 47-hub model genes?

 Discussion, page 14:

 Prediction performance and cancer types is discussed, what about "hematopoietic and lymphoid tissues"? This cancer type seems to strongly differ from other types in their drug responses and gene expression (see Figures 2 and 4). Could the authors comment on these data?

 Discussion, page 16:

 " *Nevertheless future work could benefit from the incorporation of other "omics" data .. CCLE RNA-seq dataset could allow the analysis of transcript-level ( gene isoform)". *However RNA-seq technology has a larger potential than extracting transcript isoform and allows to extract genomic (mutation, indels, gene fusion, ...) and transcriptomic events (gene expression, splice variant, non-coding RNA), could the authors enlarge their comment about this potential and their prediction method? Also what about epigenetics data to predict drug sensitivity?

I have read this submission. I believe that I have an appropriate level of expertise to confirm that it is of an acceptable scientific standard.

# Response

**Published:** 2019-02-22

**Authors:** Francisco Azuaje

**Affiliations:** Luxembourg Institute of Health (LIH), Luxembourg

**Response to** **Reviewer# 2: Therese Commes**

“The authors present an interesting work for predicting cancer cells drug responses based on " *the gene expression profile of 47 genes representing significant hubs in pan cancer transcriptomes" *from untreated cells lines. The approach is original, as they did not select a specific set of genes from drug sensitivity experiments, but proposed " *an unbiased discovery approach, motivated by the hypothesis that co-expression networkhubs encode useful information for investigating drug responsein vitro". *Next, they investigated co-expression network hubs and drug responses and validated their approach using independant data sets including cell line-drug data.

The study is well conceived and executed. The approaches used are suitable, and the description of work is adequately detailed. Data are clearly presented, and for the most part conclusions are reasonable. The method appears interesting, I think the paper would be a nice contribution that will be well-cited. Despite the difficulty of comparison, prediction performance of the model was compared to existing published methods and shows comparable results. Moreover the manuscript is well written.

The authors need to clarify on the following points:”

**Response:**

We thank the reviewer for her interest in our article and helpful feedback.

"Figure1:

It will be better to specify the type of data (microarrays?) and source (CCLE?) in input for *"transcriptomic data from untreated cell lines"."*

**Response:**

Figure 1 has been modified with the inclusion of the data type and the source as follows:

“(CCLE microarray data)”.

“Methods, page 4:

The reason why removal of genes is needed is not clear: *"Although lasso or elastic net regularizations are also suitable approaches, they would have required the estimation of additional learning parameters and the removal of genes, which were deemed biologically interesting before model training......."”*

**Response:**

We agree that, in general, the removal of genes is not required. However, note that we compared our 47-gene model with a Lasso model that consists of a reduced set of input genes. This gene selection process is embedded into the Lassso algorithm, which aims at selecting a subset of covariates with a good prediction ability. To do so, the algorithm constraints the sum of the absolute values of the coefficients. During this process, some coefficients will be set to zero and therefore are removed from the model. The elastic net, which can be seen as a compromise between Lasso and Ridge regression, also incorporates feature selection.

“Methods, page 5 (line 24):

The authors should specify what they mean by " *the original data"* which kind of files they used (raw data, gene counts ...). (line 28: RNS-seq instead of RNA-seq).”

**Response:**

In the main text, we have corrected “original data” with “RPKM data”.

We have also corrected “RNS-Seq” with “RNA-Seq”.

“Results, page 9:

Could the authors specify if drugs with nearest clusters or CI values belong to a same drug "class" or not? Is there a relationship between performance prediction and drug classes?”

**Response:**

To address the reviewer’s request, the following sentence has been added in “Result” section (page 9):

Interestingly, we observe that drugs belonging to the same drug class tend to cluster together according to their predicted (and observed) drug response values. For example, samples treated with cytotoxic drugs (e.g., Irinotecan and Topotecan) and kinase inhibitors (e.g., AZD6244 and RAF265) are closely located on the observed vs. predicted sensitivity plot (Supplementary Figure 5).

“Results, page 9:

This sentence is not clear: " *As the GDSC dataset shares "different" cell lines in common with CCLE*..."”

**Response:**

The sentence has been corrected:

“As the GDSC dataset shares cell lines in common with CCLE..."”

“Results, page 12:

" *However elastic net model required 613 genes as input features"*: Does this set include the 47-hub model genes?”

**Response:**

There are no genes in common between these models. This is now clarified in the manuscript.

Also, we note that there was a typo in this sentence: It should be 614 genes.

We have corrected this typo in the main manuscript and have changed the caption of the Supplementary Figure 13. We have also changed the URL address that contains all supplementary files (reference 23 in the main manuscript).

We include the following in the main manuscript:

“However, the elastic net model required 614 genes as input features to achieve this performance (with no genes in common with the 47 hub genes).”

“Discussion, page 14:

Prediction performance and cancer types is discussed, what about "hematopoietic and lymphoid tissues"? This cancer type seems to strongly differ from other types in their drug responses and gene expression (see Figures 2 and 4). Could the authors comment on these data?”

**Response:**

To address the reviewer’s request, the following sentence has been added:

Importantly, although the gene expression profiles of hematopoietic and lymphoid samples differ from all other samples, our 47-hub model was able to predict their responses with a relatively good accuracy (CI = 0.75).

“Discussion, page 16:

" *Nevertheless future work could benefit from the incorporation of other "omics" data. CCLE RNA-seq dataset could allow the analysis of transcript-level (gene isoform)". *However, RNA-seq technology has a larger potential than extracting transcript isoform and allows to extract genomic (mutation, indels, gene fusion, ...) and transcriptomic events (gene expression, splice variant, non-coding RNA), could the authors enlarge their comment about this potential and their prediction method? Also what about epigenetics data to predict drug sensitivity?”

**Response:**

To address the reviewer’s comment, the following text has been added:

Furthermore, there are other opportunities to be investigated such as the analysis of genomic alterations, non-coding RNAs and epigenetic markers, which may enhance or complement existing models for predicting drug sensitivity.

# Peer review: Reviewer response for version 1

**DOI:** 10.5256/f1000research.18749.r42324

**Published:** 2019-01-08

**Authors:** Elizabeth A. Coker

**Affiliations:** 1 Translational Cancer Genomics, Wellcome Trust Sanger Institute, Hinxton, UK

In this paper, Azuaje *et al*., utilise transcriptional data from the Cancer Cell Line Encyclopedia to construct gene co-expression networks and identify hubs within them. They assess the biological relevance of the hub genes, and through the use of comparatively simple linear regression-based approaches, can use gene expression of these hubs to predict drug sensitivity *in vitro*. Despite not initially aiming to predict drug sensitivity, the authors’ models are able to perform comparably to previously published approaches. Azuaje *et al*., have performed extensive and appropriate validation of their work both *in silico* and *in vitro*, including assessing the inter-platform robustness of network hubs, cross-validation of the 47-hub signature, investigating the effect of network size on hub detection, and re-implementation of their own predictive approach to allow comparison with previously published drug sensitivity predictors.   

 The paper is well-written and clear, with a thorough and precise account of the approaches used to assess the performance of the predictive model. The authors have produced a thoughtful and detailed account of their work which I enjoyed reading. The online Dr Paso resource is easy to use and has good documentation, although I have only tested it with the example datasets provided. I was unable to access the extended data via Zenodo as described in the manuscript (see comment below).

 My current recommendation is for “ **A** **pproved with reservations**”, as there are a number of points I feel should be addressed prior to approval:

 “Furthermore, only four genes in our list of hubs are known drug targets: DKK1 (Irinotecan), MYB, SPARC and TUBB6 (the latter three targeted by paclitaxel).” I disagree with this statement. I have been unable to find any evidence of irinotecan targeting the protein DKK1, apart from an entry in DGIdb that states there is an interaction of type ‘n/a’ between DKKI and irinotecan, based solely upon a paper in Oncotarget that states DDK1 does not affect sensitivity to irinotecan in two cell lines. Equally, MYB and SPARC are not targets of paclitaxel, although they are shown in the DGIdb database as interacting with paclitaxel based on little or no evidence. I recommend the authors remove this sentence from the manuscript as it is based on a misinterpretation of DGIdb results and as such is misleading.

 I was able to access the code for the Dr Paso tool via Zenodo, but not the extended data associated with this study. Please ensure this is added and the appropriate link included.

 Figure 1: Please update this figure to highlight the filtering stage between collecting transcriptomics data and building co-expression networks. Listing the number of genes present before and after filtering would also be informative.

 Figure 2a: Here, the weight of edges is used to represent the expression correlations between nodes, but at present it is very difficult to see this in the examples highlighted. A larger, higher-resolution image of the network and examples is required.

 Figure 2b: I am not familiar with the concepts of semantic space and so do not know how to interpret this plot. Consider adding extra discussion in the main text or presenting this data in another way.

 Figure 2d: This figure is currently very difficult to read and could be enlarged if 2c is concurrently decreased in size.

 Figure 3a: The density plot requires further annotation in relation to the clusters described in the manuscript, for example by arrows or circling the appropriate regions of the plot.

 Figure 4a: This figure is currently very difficult to read and could be enlarged.

 In conclusion this is an interesting paper illustrating how co-expression hubs can be used to predict drug responses *in vitro* with reasonable accuracy. The authors have clearly put a great deal of time and thought into this project and should be pleased with the resulting paper.

I have read this submission. I believe that I have an appropriate level of expertise to confirm that it is of an acceptable scientific standard, however I have significant reservations, as outlined above.

# Response

**Published:** 2019-02-22

**Authors:** Francisco Azuaje

**Affiliations:** Luxembourg Institute of Health (LIH), Luxembourg

**Response to Reviewer #1: Elizabeth A. Coker**

“In this paper, Azuaje et al., utilise transcriptional data from the Cancer Cell Line Encyclopedia to construct gene co-expression networks and identify hubs within them. They assess the biological relevance of the hub genes, and through the use of comparatively simple linear regression-based approaches, can use gene expression of these hubs to predict drug sensitivity in vitro. Despite not initially aiming to predict drug sensitivity, the authors’ models are able to perform comparably to previously published approaches. Azuaje et al., have performed extensive and appropriate validation of their work both in silico and in vitro, including assessing the inter-platform robustness of network hubs, cross-validation of the 47-hub signature, investigating the effect of network size on hub detection, and re-implementation of their own predictive approach to allow comparison with previously published drug sensitivity predictors.

The paper is well-written and clear, with a thorough and precise account of the approaches used to assess the performance of the predictive model. The authors have produced a thoughtful and detailed account of their work which I enjoyed reading. The online Dr Paso resource is easy to use and has good documentation, although I have only tested it with the example datasets provided. I was unable to access the extended data via Zenodo as described in the manuscript (see comment below).

My current recommendation is for “Approved with reservations”, as there are a number of points I feel should be addressed prior to approval:”

**Response:**

We thank the reviewer for her interest in our article and helpful feedback.

 “Furthermore, only four genes in our list of hubs are known drug targets: DKK1 (Irinotecan), MYB, SPARC and TUBB6 (the latter three targeted by paclitaxel).” I disagree with this statement. I have been unable to find any evidence of irinotecan targeting the protein DKK1, apart from an entry in DGIdb that states there is an interaction of type ‘n/a’ between DKKI and irinotecan, based solely upon a paper in Oncotarget that states DDK1 does not affect sensitivity to irinotecan in two cell lines. Equally, MYB and SPARC are not targets of paclitaxel, although they are shown in the DGIdb database as interacting with paclitaxel based on little or no evidence. I recommend the authors remove this sentence from the manuscript as it is based on a misinterpretation of DGIdb results and as such is misleading.”

**Response:**

We agree that this sentence is not accurate. As requested, we have adapted it as follows:

  

We did not find validated evidence that our hub list contains known drug targets. Using DGIdb, we found potential associations between 4 hubs and 2 drugs: DKK1 (with Irinotecan), MYB, SPARC and TUBB6 (the latter three with paclitaxel). However, these associations cannot be interpreted as drug-target interactions and require further investigation.

“I was able to access the code for the Dr Paso tool via Zenodo, *but not the extended data associated with this study*. Please ensure this is added and the appropriate link included.”

**Response:**

We confirm that the extended datasets are available in Zenodo, and they can be accessed via the web link included in reference 23.

“Figure 1: Please update this figure to highlight the filtering stage between collecting transcriptomics data and building co-expression networks. Listing the number of genes present before and after filtering would also be informative.”

**Response:**

Change made, as requested.

“Figure 2a: Here, the weight of edges is used to represent the expression correlations between nodes, but at present it is very difficult to see this in the examples highlighted. A larger, higher-resolution image of the network and examples is required.”

**Response:**

The figure has been modified to improve its clarity. The edges are represented with a white-to-grey gradient: the darker the edge, the higher the correlation.

Although the edges connected to the non-hub node are expected to be more difficult to visualize because they are weaker, the modified figure now shows a better contrast between the hub and non-hub examples in terms of their corresponding edges.

The figure caption has also been adapted to improve clarity:

“(A) Snapshot of a (fully connected) weighted gene correlation network from untreated cell lines. Nodes and edges representing genes and their correlations respectively. Network hubs and non-hubs are colored in green and black respectively. Nodes are connected by edges, which are depicted in a white-to-grey gradient (the darker the edge, the higher the correlation). A zoom-in view of examples of hub and non-hub nodes reveals that the hub node has more edges with higher weights compared to the non-hub node.”

“Figure 2b: I am not familiar with the concepts of semantic space and so do not know how to interpret this plot. Consider adding extra discussion in the main text or presenting this data in another way.”

**Response:**

We have expanded the caption of the figure with additional text to facilitate interpretation, as follows:

“(B) Graphical summary of (non-redundant) Gene Ontology terms statistically over-represented in the list of 47 hub genes. Significant Biological Processes terms, represented as bubbles, are projected onto a scatterplot using REVIGO (33). Terms sharing common ancestors in the Gene Ontology database are close together; leading to a cluster of GO terms characterizing highly related biological annotations. To facilitate visualization, only a small selection of terms are labeled on the figure. Color and size indicates the term’s level of statistical enrichment in our list of hubs and frequency in the GO database respectively.”

“Figure 2d: This figure is currently very difficult to read and could be enlarged if 2c is concurrently decreased in size.”

**Response:**

Changes made, as recommended.

“Figure 3a: The density plot requires further annotation in relation to the clusters described in the manuscript, for example by arrows or circling the appropriate regions of the plot.”

**Response:**

Changes made, as requested.

“Figure 4a: This figure is currently very difficult to read and could be enlarged.”

**Response:**

Figure has been enlarged, as requested.

“In conclusion this is an interesting paper illustrating how co-expression hubs can be used to predict drug responses *in vitro* with reasonable accuracy. The authors have clearly put a great deal of time and thought into this project and should be pleased with the resulting paper.”
